# DNA barcodes enable higher taxonomic assignments in the Acari

**DOI:** 10.1038/s41598-021-95147-8

**Published:** 2021-08-05

**Authors:** Monica R. Young, Jeremy R. deWaard, Paul D. N. Hebert

**Affiliations:** 1 Centre for Biodiversity Genomics, University of Guelph, 50 Stone Road East, Guelph, ON N1G 2W1 Canada; 2 Department of Integrative Biology, University of Guelph, 50 Stone Road East, Guelph, ON N1G 2W1 Canada; 3 School of Environmental Sciences, University of Guelph, 50 Stone Road East, Guelph, ON N1G 2W1 Canada

**Keywords:** DNA sequencing, Next-generation sequencing, Classification and taxonomy, Data processing, Genetic markers

## Abstract

Although mites (Acari) are abundant in many terrestrial and freshwater ecosystems, their diversity is poorly understood. Since most mite species can be distinguished by variation in the DNA barcode region of cytochrome *c* oxidase I, the Barcode Index Number (BIN) system provides a reliable species proxy that facilitates large-scale surveys. Such analysis reveals many new BINs that can only be identified as Acari until they are examined by a taxonomic specialist. This study demonstrates that the Barcode of Life Datasystem’s identification engine (BOLD ID) generally delivers correct ordinal and family assignments from both full-length DNA barcodes and their truncated versions gathered in metabarcoding studies. This result was demonstrated by examining BOLD ID’s capacity to assign 7021 mite BINs to their correct order (4) and family (189). Identification success improved with sequence length and taxon coverage but varied among orders indicating the need for lineage-specific thresholds. A strict sequence similarity threshold (86.6%) prevented all ordinal misassignments and allowed the identification of 78.6% of the 7021 BINs. However, higher thresholds were required to eliminate family misassignments for Sarcoptiformes (89.9%), and Trombidiformes (91.4%), consequently reducing the proportion of BINs identified to 68.6%. Lineages with low barcode coverage in the reference library should be prioritized for barcode library expansion to improve assignment success.

## Introduction

The Acari (mites) are very abundant and species-rich in many terrestrial and freshwater habitats^1^. They comprise nearly 55,000 described species from six orders belonging to two potentially diphyletic superorders (Acariformes, Parasitiformes) but are often treated as a single group^2,3^. Most species are ascribed to the acariform orders Trombidiformes (25,146) and Sarcoptiformes (16,630), with smaller contributions from the parasitiform orders including Mesostigmata (12,017), Ixodida (900), Holothyrida (35), and Opilioacarida (35)^1^. Despite their prevalence, mite diversity remains poorly understood because their small size and often cryptic morphology pose challenges to identification^4,5^. However, most mite species can be distinguished by sequence variation in the 648 bp barcode region of the mitochondrial cytochrome *c* oxidase I (COI) gene^6–8^. Because operational taxonomic units delineated by the Barcode Index Number (BIN) system^9^ are an effective proxy for mite species^10^, DNA barcoding enables large-scale surveys that will undoubtedly reveal many novel taxa. For example, a recent study concluded that more than 20,000 mite BINs await detection in Canada alone^11^. Since this nation is thought to host about 1% of the world’s fauna^12^, more than two million mite BINs likely await registration. While just 15,000 are currently represented on BOLD, the Barcode of Life Datasystem^13^, these records do provide coverage for all six orders and for more than half of all known families (278/533; see “Methods”). In fact, there are many records for the more speciose (≥ 20 spp.) families with North American, Holarctic, or cosmopolitan distributions. The capacity to assign newly encountered BINs to these higher taxonomic ranks based on their barcode sequence alone can provide biological context for taxa that would otherwise lack such information^11^. However, no previous study has tested the accuracy of assignments to higher taxonomic ranks based solely on DNA barcode sequences from mites.

DNA barcodes have been shown to enable accurate placements to higher-level taxa in other animal lineages including amphibians and reptiles^14,15^, moths^16^, and spiders^17^. Although tree-based methods have been successful^16,18^, only queries nested within a monophyletic clade can be confidently identified with this approach^19^. Since COI is not ideal for resolving deep nodes^20,21^, methods reliant on tree topology for higher taxonomic assignments using DNA barcodes will often be constrained. By contrast, distance-based methods can enable assignments even in the absence of monophyly using diagnostic thresholds^19^. This approach often outperforms tree-based methods in assigning unknown queries to a higher taxon^14,16,22^. However, the performance of distance-based methods varies among taxonomic groups and with taxon coverage^16,17^. The length of the query sequence also affects assignment success^23^. Accordingly, the distance-based thresholds adopted for assignments to higher taxonomic ranks are probably lineage specific and dependent on library completeness and sequence length. While the first two factors limit the adoption of a standard threshold for the animal kingdom, the last impacts the analysis of data from metabarcoding studies because they typically rely on short sequences^24^.

This study evaluates the accuracy and precision of higher-taxon assignments based on DNA barcodes for a hemi-continental fauna—the Acari of Canada. These taxa have received more intensive DNA barcode analysis than any other national fauna^11^. The current reference library provides coverage for all four orders and for 60% (188) of the families known from Canada^11^. While this comprises just a third of the world’s mite families, most of the speciose (≥ 20 spp. described) families known from soil and leaf litter in North America are represented^4,25^. The library also includes coverage for many of the Nearctic families associated with plants, birds, and freshwater habitats. Datasets based on two sequence lengths and three levels of sequence coverage for higher taxa were used to evaluate the accuracy and precision of assignments made using a distance-based identification method: BOLD ID^13^. From this analysis, thresholds for the identification of newly encountered mite BINs to an order and family were obtained, and the implications of their use at broader geographic scales are discussed.

## Methods

### Construction of six data sets

Datasets were constructed based upon a well-curated reference library for Canadian mites^11^. Identification success was compared for three levels of library completeness by systematically reducing sequence coverage for higher taxa. The most complete dataset was assembled by selecting the longest sequence (generally 658 bp) with the fewest ambiguous nucleotides for each BIN identified to a family or lower level (BIN dataset; 7021 sequences). These sequences were then aligned by amino acid translation with MUSCLE in MEGA 6.06^26^ to allow the construction of a matrix of uncorrected nucleotide p-distances with pairwise deletion using the ‘APE’ package^27^ in R version 3.5.0^28^. This substitution model was chosen to allow comparison with BOLD ID which utilizes p-distances in downstream analyses. This matrix was used to hierarchically cluster the sequences by single-linkage to partition them into clusters with at least 5% nucleotide divergence. Silhouette scores were then computed for each sequence using the ‘cluster’ package^29^ in R as a measure of its centrality within its cluster^30^. The BIN sequence with the highest silhouette score was selected to represent each 5% cluster (DIV5 dataset; 5182 sequences). The least complete dataset (DIV10 dataset; 3948 sequences) was constructed by selecting a representative sequence from each cluster with at least 10% nucleotide divergence using the same approach but with the DIV5 dataset as a starting point.

All sequences in the BIN dataset were at least 500 bp in length with less than 1% ambiguous bases. Those with sequence coverage outside the 658 bp barcode segment were trimmed to only include the target region. To simulate the shorter sequence data recovered with standard metabarcoding protocols, sequences in the three datasets were trimmed to the 463 bp fragment of COI generated by the AncientLepF3 and LepR1/HCO2198 primer pairs^31^ to produce three additional datasets (tBIN, tDIV5, tDIV10). This amplicon has demonstrated low taxonomic bias and high BIN recovery when employed for metabarcoding studies on mock arthropod communities and provides strong species resolution in many arthropod groups^31,32^. Consequently, this fragment provides a good opportunity to evaluate the impact of reduced sequence length on assignments to higher taxonomic categories.

Family-level taxonomy followed Zhang^25^ with the following exceptions. The Nenteriidae were included in the Trematuridae and the Uroactiniidae in the Urodynichidae following Beaulieu et al.^3^. The Dithinozerconidae were treated as distinct from the Trachytidae, and a potentially undescribed mesostigmatan family (MRY1)^11^ was included. The recently described family Dytiscacaridae^33^ was also included while the Nalepellidae were treated as a separate family from the Phytoptidae following Chetverikov et al.^34^. The Erythracaridae were considered as separate from the Anystidae following Pepato and Klimov^35^ and the Eutrombidiidae from the Microtrombidiidae (C. Welbourn, pers. comm.). Two Oribatida families have been recognized as junior homonyms so we adopted their correct names: Compactozetidae (= Cepheidae), and Punctoribatidae (= Mycobatidae). Although some of these taxa were not represented in this study, they were included in the tally of recognized mite families.

### Intra- and intertaxon divergence

Patterns of intra- and intertaxon divergence among mite orders and families were evaluated for each dataset by calculating the maximum distance within and minimum distance between each taxon from the p-distance matrices using ‘SPIDER’^36^ in R. Although interfamily distances were calculated for monotypic families, they necessarily lacked an intrafamily distance value. The effects of sequence coverage, sequence length, and order assignment on divergence patterns were evaluated by analysis of variance (ANOVA) both including and excluding outliers detected using the ‘rstatix’^37^ R package. When significant differences were revealed by ANOVA, all pairwise comparisons of divergence were evaluated using Tukey’s HSD test in R.

Divergence values were examined to ascertain if there was a ‘barcode gap’ separating maximum intra- and minimum interfamily p-distance. Additionally, the relationship between the maximum divergence value for a family and its number of component BINs was examined using linear, logarithmic, and asymptotic regression models in R. The best model was selected by evaluating Akaike’s information criterion (AIC) and residual standard error (RSE). The same analyses were not conducted at the order level because of low sample size (n = 4).

### Identifier performance and threshold selection

BOLD ID’s performance was evaluated for each dataset using a modified leave one out cross validation method where each sequence was queried against ‘All Barcode Records’ on BOLD [April 2020] using the identification engine. The resultant taxonomic assignment was recorded and categorized as a correct or incorrect match at the order and family level along with the associated similarity score and bp overlap with the top BOLD ID hit after excluding self-matches, those lacking a family identification, and those with limited bp overlap (50%). To prevent query sequences from matching to other members of their own BIN, 5%, and 10% divergence cluster, the closest match exceeding 2%, 5%, and 10% divergence from the query was considered the top hit for the BIN, DIV5, and DIV10 datasets respectively. The proportion of queries correctly identified to an order and family was summarized across all Acari and for each order separately. Differences in the proportion of correct assignments with sequence coverage, length, and order were assessed with Pearson's chi-square tests.

The ‘All Barcode Records’ library comprises more than 7.6 million COI sequences from 32 animal phyla including 130,000 sequences representing 15,313 BINs from 278 families and six orders of mites with the same ordinal and family-level taxonomic system employed in this study. Although BOLD’s records are curated, some taxonomic assignments are undoubtedly incorrect, reflecting identification errors or contaminated sequences. To ensure no major errors were present prior to analysis, we constructed a Kimura-2-Parameter Neighbor-Joining tree using BOLD’s Taxon ID Tree function including one representative from each mite BIN. The tree was inspected for long branches and incongruent taxonomy within clusters. Suspect BINs were queried against ‘All Barcode Records’ using BOLD ID to reveal cases of potential contamination or misidentification. This review revealed 39 (0.2%) errors which were excluded from the reference library. Possible errors in BOLD’s non-mite records were evaluated post hoc by querying the sequences of all top hits belonging to a non-mite order and examining their closest matches.

Receiver operating characteristic (ROC) curves were used to examine the true positive rate (TPR) and false positive rate (FPR) across all hypothetical thresholds using the ‘pROC’ package^38^ in R. TPR is the ratio of true positives (TP; correctly identified sequences with similarity scores above the threshold) to the total number of correctly identified sequences including TPs and false negatives (FN; correctly identified sequences falling below the similarity threshold). Conversely, FPR is the ratio of false positives (FP; incorrectly identified sequences above the similarity threshold) to the total number of incorrectly identified sequences including FPs and true negatives (TN; incorrectly identified sequences with similarity scores below the threshold). BOLD ID’s performance was compared across all datasets and for each order by estimating the area under each curve (AUC) and analyzing differences using the DeLong et al.^39^ method as implemented by ‘pROC’. AUC ranges from 0 to 1 where higher values indicate good performance (1 = all assignments are correct) and lower values indicate poor performance (0 = all assignments are incorrect), while 0.5 indicates that the identifier performance is no better than chance.

**Figure 1:**
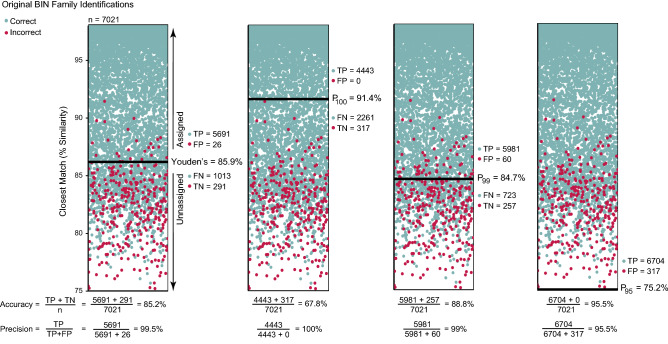
Schematic representation of the protocol employed for sequence assignment to a family using Youden’s J statistic and three precision-based thresholds (P_100_, P_99_, P_95_) for full-length BIN sequences including calculations of accuracy and precision. BINs whose closest match fall above the threshold (indicated by a solid black line) are assigned to a family and can include both true positive (TP) and false positive (FP) matches. BINs whose closest match fall below the threshold are left unassigned and represent both true negative (TN) and false negative (FN) matches.

Accuracy and precision were calculated for all hypothetical thresholds for each dataset and for each order using ‘pROC’. Accuracy represents the proportion of TP and TN compared to the total number of sequences [(TP + TN)/n] while precision measures the proportion of sequences with similarity scores above the threshold that are correctly assigned [TP/(TP + FP)]. Accuracy and precision were also calculated for thresholds of order- and family-level identification determined by Youden’s J statistic^40^, reflecting the upper-left portion of ROC curves where TPR is maximized and FPR is minimized. Although this method is commonly used for threshold selection in ROC analyses, it weights the true negative rate (TNR = 1 − FPR) and TPR equally, allowing a variable amount of error in the positive predicted class^41^. If false positives have serious impacts on data interpretation, precision-based thresholds which specify an acceptable level of error in the positive predicted class may be more appropriate for application. Therefore, accuracy was also estimated for thresholds determined using three precision-based criteria allowing 0% (P_100_), 1% (P_99_), and 5% (P_95_) error in the positive predicted class (Fig. 1). If precision across all hypothetical thresholds was higher than defined by a threshold criterion (i.e. precision > 95%), that threshold (i.e. P_95_) was set to the minimum similarity score recorded.

All values are reported for the BIN dataset, unless otherwise specified.

## Results

The BIN dataset included 7021 COI sequences derived from Canadian specimens that belonged to 189 families and four orders with an average coverage of 37 sequences (SD = 64) per family (Supplementary Table S1). Most families belonged to the Sarcoptiformes (89), followed by the Trombidiformes (61), the Mesostigmata (38), and Ixodida (1). Just 27 families (14%) were represented by a single sequence, most of which are thought to be species poor (< 10) in Canada or restricted to weakly sampled habitats (e.g., vertebrate hosts). The sequences had an average length of 647 bp (SD = 24, range = 504–691 bp) with less than 1% ambiguous bases, and 74% were full length (≥ 648 bp). By comparison, the trimmed sequences averaged 453 bp (SD = 21, range = 338–466 bp), 74% of which had full coverage for the 463 bp target region (a few sequences were 466 bp reflecting their possession of a 3 bp insertion). The number of sequences was reduced to 5182 in the DIV5 (${\overline{\text{x}}}$  = 27 seq/family, SD = 44) and to 3948 in the DIV10 datasets (${\overline{\text{x}}}$ = 21 seq/family, SD = 30). While each dataset included coverage for all 189 families, the incidence of singleton families rose in the DIV5 (20%) and DIV10 (21%) datasets.

### Intra- and intertaxon divergence

**Figure 2:**
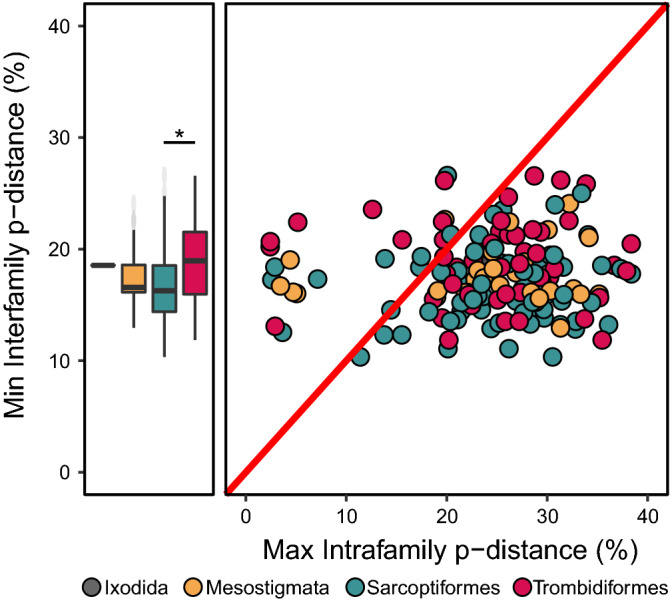
The distribution of maximum intrafamilial and minimum interfamilial DNA barcode divergences (p-distance) for the full-length (${\overline{\text{x}}}$ = 647 bp) sequences from representatives of 7021 BINs and 162 families of mites. Points above the red line indicate families whose intrafamily divergence is less than the distance to their nearest neighbor, while those below the line do not meet this criterion. Outliers are shown with reduced opacity. Significant differences in the distribution of minimum interfamilial divergence for two of the four major orders of mites are denoted with an asterisk (*).

**Figure 3:**
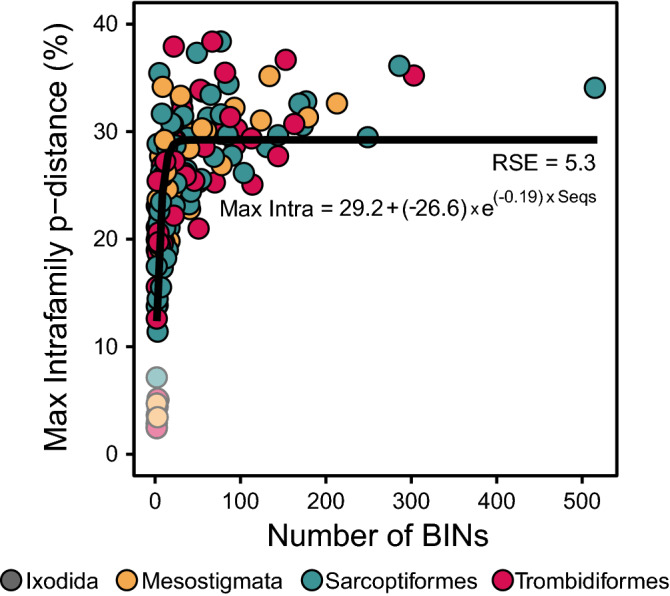
The relationship between maximum intrafamilial divergence values and the number of full-length (${\overline{\text{x}}}$ = 647 bp) DNA barcode sequences analyzed for 162 families from four orders of mites. Outliers are shown with reduced opacity.

Maximum intraorder p-distances were high (${\overline{\text{x}}}$ = 39.3%, SD = 10.7) and did not differ with sequence length (ANOVA, p = 0.8) or coverage (ANOVA, p = 0.9). Intrafamilial p-distances were also high (${\overline{\text{x}}}$ = 24.5%, SD = 8.0; Fig. 2) and did not differ with sequence length (ANOVA, p = 0.2), coverage (ANOVA, p = 0.1), or among the four orders (ANOVA, p = 0.5). However, maximum intrafamilial p-distance was significantly correlated to the number of BINs in a family (p < 0.0001) and reached an asymptote at approximately 29% p-distance while just seven BINs were required to reach half the asymptotic divergence (Fig. 3). Although linear and log models also fit the data well (p < 0.0001), AIC and RSE scores were lowest for the asymptotic model (Supplementary Table S2).

The mean of the minimum distance values between BINs in different orders (${\overline{\text{x}}}$ = 17.9%, SD = 0.7) was significantly less than the mean maximum distance among BINs in an order (${\overline{\text{x}}}$ = 39.3%, SD = 10.7; ANOVA p = 0.007). These patterns were unaffected by sequence length (ANOVA, p = 0.4) or coverage (ANOVA, p = 0.7). The mean minimum distance value between BINs in different families (${\overline{\text{x}}}$ = 17.6%, SD = 3.5) was also lower than the mean maximum distances (${\overline{\text{x}}}$ = 24.5%, SD = 8.0) within families (ANOVA, p < 0.00001; Fig. 2). Interfamilial divergences were unaffected by sequence length (ANOVA, p = 0.5) or coverage (ANOVA, p = 0.5). However, differences were present between two of the four orders (ANOVA, p < 0.001; Tukey’s, p < 0.001) with minimum interfamilial p-distances significantly higher for the Trombidiformes (${\overline{\text{x}}}$ = 19.0%, SD = 3.7) than for the Sarcoptiformes (${\overline{\text{x}}}$ = 16.7%, SD = 3.4; Fig. 2) even after removing outliers.

### Identifier performance and threshold selection

#### Order-level identification

**Figure 4:**
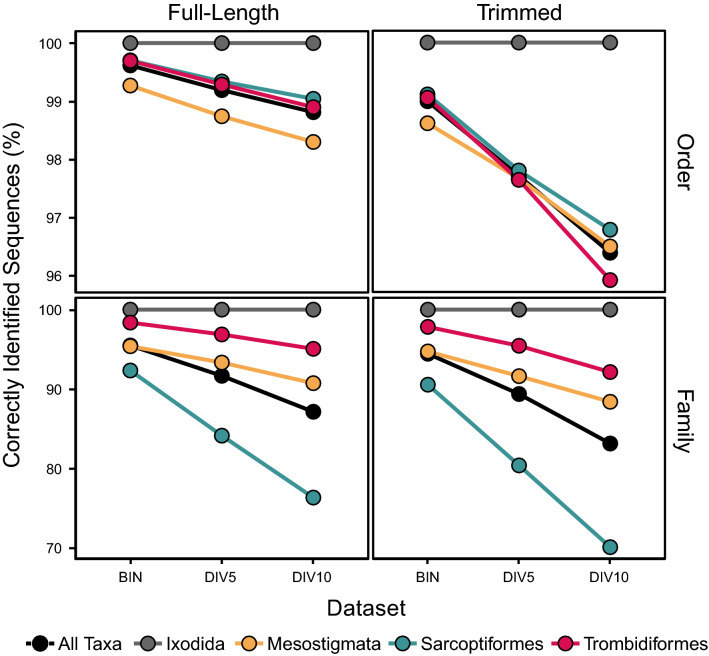
The percentage of full-length (${\overline{\text{x}}}$ = 647 bp) and trimmed (${\overline{\text{x}}}$ = 453 bp) DNA barcode sequences assigned to the correct order and family by BOLD ID for datasets representing three levels of sequence coverage: Barcode Index Numbers (BIN), 5% sequence clusters (DIV5), and 10% sequence clusters (DIV10). Data is shown for all taxa combined and for each order separately.

Most (> 96.4%) taxa were assigned to the correct order (Fig. 4). Success declined slightly with reduced sequence coverage (χ^2^, p < 0.00001) and length (χ^2^, p < 0.001) in all datasets, but did not differ among the four orders (χ^2^, p = 0.15 to p = 0.9; Supplementary Table S3). The number of misassigned sequences ranged from 27–143 across the six datasets. Sequences misassigned in the more complete datasets were also misassigned in the less complete ones, but few sequences were misassigned in both the full-length and trimmed datasets (10–18% of total). A total of 161 sequences were misassigned in at least one of the six datasets including 48 full length and 142 trimmed sequences. Similarity scores for misassignments ranged from 74.5 to 86.6% (${\overline{\text{x}}}$ = 80.1%, SD = 2.9) for full-length and from 64.3%-90.7% (${\overline{\text{x}}}$ = 79.7%, SD = 3.3) for trimmed sequences. Misassigned sequences in the full-length datasets (range = 27–47) were occasionally placed in an incorrect mite order (Ixodida, Sarcoptiformes) but were mostly placed in other arthropod orders (Araneae, Coleoptera, Decapoda, Diptera, Hemiptera, Hymenoptera, Lepidoptera). However, one was most closely related (75.4%) to a vesper bat (*Myotis velifer*) sequence originating from GenBank (MF143499). Misassigned sequences in the trimmed datasets (range = 70–141) were also generally placed in other arthropod orders (Araneae, Diptera, Coleoptera, Hemiptera, Hymenoptera, Lepidoptera, Orthoptera) but a higher proportion of the misassigned sequences (range = 33–46%) were most closely related to one of three chordate sequences originating from GenBank including the vesper bat, a domestic goat (KJ192226), and a human (KJ937463) sequence. In fact, the highest ranked incorrect order-level assignment was returned for a trimmed Eupodidae sequence that possessed 90.7% similarity to a bat sequence. Morphological inspection confirmed the specimen was a eupodid, and the bat sequence was also valid as its closest neighbours were all members of the genus *Myotis*. Careful examination of the other misassignments indicated that just six arose from errors in the reference library. For example, an 82.7% match between a phoretic mite (Pygmephoridae) and a beetle (Hydrophilidae) revealed that the supposed beetle sequence actually derived from a pygmephorid mite since all close matches to it belonged to pygmephorids rather than beetles. Examination of the source beetle confirmed that it was carrying several pygmephorid inquilines. Five more misassignments likely also derived from errors in the reference database, four involving nearest-neighbor matches to a supposed insect BIN (1 Coleoptera, 1 Diptera, 2 Hymenoptera) and one to a supposed spider BIN. In each case, the supposed insect/spider was deeply embedded in a mite clade, suggesting the sequence in each case derived from a phoretic heterostigmatid mite (Pygmephoridae, Tarsonemidae) rather than its insect/spider host. However, these cases could not be confirmed because the mite specimens were unavailable for morphological analysis.

**Figure 5:**
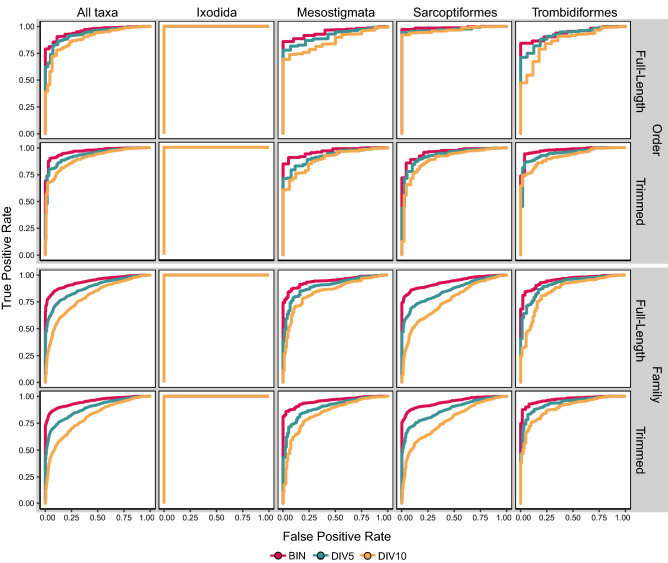
Receiver operating characteristic (ROC) curves for ordinal and family-level identifications of full-length (${\overline{\text{x}}}$ = 647 bp) and trimmed (${\overline{\text{x}}}$ = 453 bp) DNA barcode sequences by BOLD ID. ROC curves are shown for all taxa combined and for each order separately for datasets with three levels of sequence coverage: Barcode Index Numbers (BIN), 5% sequence clusters (DIV5), and 10% sequence clusters (DIV10).

**Figure 6:**
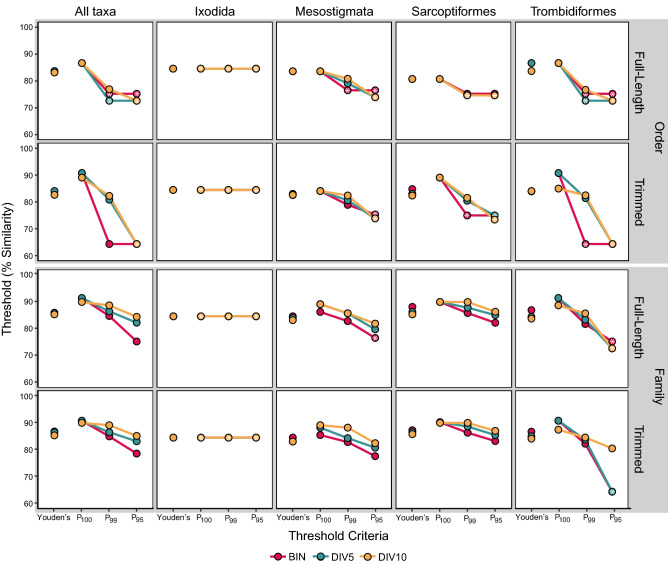
Ad hoc sequence similarity thresholds for the ordinal and family-level identification of full-length (${\overline{\text{x}}}$ = 647 bp) and trimmed (${\overline{\text{x}}}$ = 453 bp) mite DNA barcode sequences by BOLD ID. Thresholds are shown for all taxa combined and for each order separately at three levels of sequence coverage: Barcode Index Numbers (BIN), 5% sequence clusters (DIV5), and 10% sequence clusters (DIV10). Thresholds were estimated using Youden’s J statistic and three precision-based criteria allowing 0% (P_100_), 1% (P_99_) and 5% (P_95_) error in accepted identifications.

The corresponding ROC curves generally reached high TPR (> 75%) at relatively low FPR (< 5%) with higher AUCs than expected by chance (Fig. 5). The AUCs did not differ with sequence length (DeLong, p = 0.3 to p = 0.8) but were significantly higher for the BIN dataset than for DIV10 (DeLong, p = 0.004) and declined with reduced sequence coverage in the trimmed datasets (DeLong, p < 0.0001 to p = 0.02; Supplementary Table S4). The AUC for Sarcoptiformes was significantly higher than for Mesostigmata and Trombidiformes in all three of the untrimmed datasets (DeLong, p < 0.00001 to p = 0.02). Youden’s threshold (83.6%) assigned 90.1% of the BINs to an order with just three incorrect assignments (i.e. false positives) resulting in high precision (100%) and accuracy (90.4%; Supplementary Table S5). Youden’s thresholds were similar regardless of sequence length or coverage (range = 82.6–84.0%; Fig. 6) and allowed a maximum of 21 incorrect assignments (including 1 from monotypic families). However, accuracy declined with reduced coverage (e.g., DIV5 = 82.6%, DIV10 = 77.9%) despite high precision (≥ 99.8%) as fewer sequences were assigned from DIV5 (81.9%) and DIV10 (77.0%) than from the BIN dataset. Youden’s threshold also varied among the orders and was highest for the Trombidiformes (86.7%), followed by the Ixodida (84.6%), Mesostigmata (83.6%), and the Sarcoptiformes (80.7%). These thresholds assigned 89.2% of the BINs to an order with moderate accuracy (> 84.3%) and high precision (100%).

**Figure 7:**
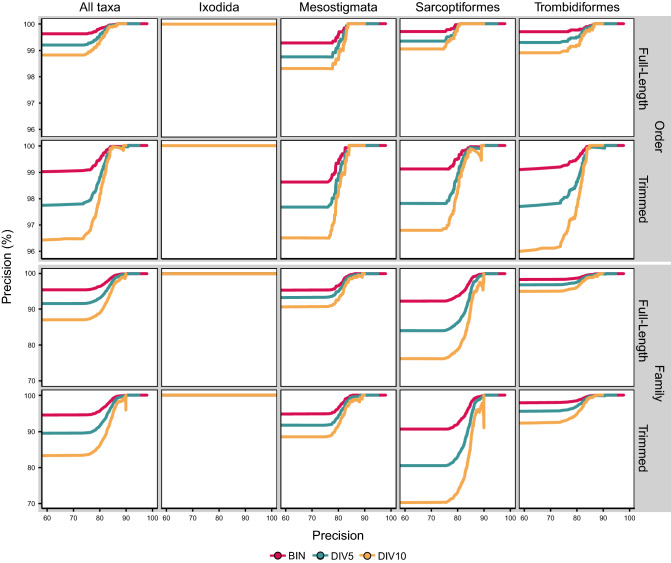
The precision of order and family-level identifications based on full-length (${\overline{\text{x}}}$ = 647 bp) and trimmed (${\overline{\text{x}}}$ = 453 bp) mite DNA barcode sequences by BOLD ID across all hypothetical similarity thresholds. Precision is shown for all taxa combined and for each order separately for datasets with three levels of sequence coverage: Barcode Index Numbers (BIN), 5% sequence clusters (DIV5), and 10% sequence clusters (DIV10).

The precision of order-level identification was generally high and exceeded 95% at all hypothetical thresholds (Fig. 7). As a consequence, the P_95_ thresholds for order-level identification were set to the minimum similarity scores recorded for all datasets and for each order (Fig. 6). Since the P_95_ thresholds enabled the assignment of all sequences, accuracy remained high (> 95%) despite the inclusion of incorrect assignments (range = 27–143 incorrect, 2–6 from monotypic families; Supplementary Table S5). Although precision also exceeded 99% at all hypothetical thresholds in the BIN, tBIN, and DIV5 datasets, the P_99_ thresholds typically increased with reduced sequence coverage and length (Fig. 6) allowing a maximum of 70 incorrect assignments (1 from monotypic). Accuracy also declined with reduced sequence coverage and length (e.g., DIV10 = 98.2%, tDIV10 = 84.8%) as P_99_ assigned fewer sequences to order (e.g., DIV10 = 99.0%, tDIV10 = 82.8%). Precision reached 100% (P_100_) at 86.6% similarity, resulting in the identification of 78.6% of all BINs with 78.0% accuracy. However, P_100_ varied by order as precison reached 100% at 80.7% similarity for Sarcoptiformes, 83.6% for Mesostigmata, 86.4% for Ixodida, and 86.6% for Trombidiformes. These order-specific thresholds increased the proportion of identified BINs to 89.2% with 89.6% accuracy.

#### Family-level identification

The proportion of sequences correctly identified to a family was significantly lower than for the order level in all datasets (χ^2^, p < 0.00001; Fig. 4). As expected, success rates were highest for the BIN dataset (95.5%) and decreased with reduced sequence coverage (χ^2^, p < 0.00001) to a low of 87.1% for DIV10. Success was also lower for each of the trimmed datasets than for its untrimmed counterpart (χ^2^, p < 0.00001 to p = 0.005). The proportion of correctly identified sequences varied significantly among orders (χ^2^, p < 0.00001) with the highest success for Ixodida (100%), followed by Trombidiformes (98.4%), Mesostigmata (95.4%), and Sarcoptiformes (92.3%; Fig. 4). While 2–3 sequences from monotypic families were among the highest ranked (≥ 88% similarity) misidentifications, most involved Astigmatina (Sarcoptiformes) sequences that matched most closely to representatives of other astigmatid families. However, highly ranked family-level misassignments also included Ascidae (Mesostigmata), Oribatida (Sarcoptiformes), Hydrachnidia and Smaridiidae (Trombidiformes) sequences, as well as trimmed sequences of Eupodidae (Trombidiformes), Blattisociidae and Pachylaelapidae (Mesostigmata).

Family ROC curves demonstrated significantly higher AUC than expected by chance (Fig. 5), reaching high TPR (> 75%) at relatively low FPR (< 10%). AUC declined with reduced sequence coverage (DeLong, p < 0.00001), but was not impacted by sequence length (DeLong, p = 0.06 to p = 0.9). The AUC for Sarcoptiformes were significantly lower than those for Mesostigmata and Trombidiformes in the DIV5 and DIV10 datasets (DeLong, p < 0.001 to p = 0.009), while the AUC for Trombidiformes was significantly higher than for those for Mesostigmata and Sarcoptiformes in the tDIV5 and tDIV10 datasets (DeLong, p < 0.00001 to p = 0.02). Youden’s threshold (85.9%) assigned 81.4% of the BINs to a family with 26 incorrect assignments resulting in high precision (99.5%) but lower accuracy (85.2%; Supplementary Table S5). Youden’s thresholds were similar regardless of sequence length or coverage (range = 85.3–86.7%) allowing a maximum of 88 incorrect assignments (7 from monotypic; Fig. 6). However, accuracy declined with reduced coverage (e.g., DIV5 = 73.8%, DIV10 = 63.2%) despite high precision (≥ 96.8%) as fewer sequences were assigned for DIV5 (67.1%) and DIV10 (53.7%) than for the BIN dataset. Youden’s thresholds also varied among orders and were typically highest for Sarcoptiformes (88.1%), followed by Trombidiformes (86.9%), Ixodida (84.6), and Mesostigmata (84.6%; Fig. 6). These thresholds assigned 80.4% of the BINs to a family with high precision (> 99.7%) and moderate accuracy (> 81%; Supplementary Table S5).

The precision of family-level identification exceeded 95% at all hypothetical thresholds for the BIN dataset (Fig. 7). As a consequence, the P_95_ threshold for this dataset was set to the minimum similarity output recorded for all mites (75.2%) and for each order except Sarcoptiformes (82.1%). Accuracy was high (99.5%) even though all 317 (16 monotypic) incorrect family assignments were allowed. By contrast, the P_99_ threshold (84.7%) assigned fewer BINs (86%) than P_95_ (100%) but just 1% of assignments were incorrect (60 total, 3 from monotypic). The P_99_ thesholds varied by order and were highest for the Sarcoptiformes (85.8%), followed by Ixodida (84.6%), Mesostigmata (82.8%), and Trombidiformes (81.7%). Although the P_99_ and P_95_ thresholds were similar regardless of sequence length, they increased with lower sequence coverage, consequently reducing the number of false positives to just 7 (2 from monotypic), but also limiting the proportion of assigned sequences (18.2%), in the DIV10 dataset with P_99_. The most strict thresholds (P_100_) were similar for all datasets (≈91%), but the proportion of assigned sequences declined with sequence coverage (e.g., BIN = 63.3%, DIV10 = 2.9%), as did accuracy accuracy (e.g., BIN = 67.8%, DIV10 = 15.8%). The P_100_ thresholds also differed among the four orders, and together enabled family assignments for 68.6% of the BINs. They were highest for the Trombidiformes (91.4%), followed by Sarcoptiformes (89.9%), Mesostigmata (86.2%), and Ixodida (84.6%).

## Discussion

This study provides the first evaluation of the capacity of DNA barcodes to assign mite BINs to higher taxonomic categories using the Canadian fauna as a test case. Analysis of more than 7000 BINs from 189 families and four orders revealed that maximal p-distances reached asymptotic values of ≈ 40% for members of an order and ≈ 30% for members of a family, reflecting the saturation of nucleotide substitution. Similar asymptotic COI divergences in salamanders (≈ 26% max K2P distance) and frogs (≈ 32% max K2P distance) were also linked to saturation^14^. Simple distance metrics (e.g., p-distance) underestimate true divergence when substitutions are saturated^42^ causing species in distantly related clades to possess similar divergences. For example, in the present study, divergences among members of an order often exceeded the minimum divergence to a BIN in a different order, and the same pattern also occurred at the family level. Such cases might reflect problems in current taxonomic systems (over-lumping, over-splitting, paraphyly, polyphyly), but are more likely to reflect the saturation of nucleotide substitutions at deep nodes^43^. More complex models of nucleotide substitution can improve divergence estimates but did not improve identification success in the present situation (data not shown). These cases of misassignment can be reduced and eventually eliminated by expanding parametrization of the reference library. The level of parameterization required to achieve accurate assignments can only be validated empirically. However, the present reference library which was based on less than 1% of the estimated mite diversity (7 K of 2 M species) performed surprisingly well as the closest match for most BINs belonged to the correct order (> 99%) and family (> 90%).

An earlier analysis of nearly 30,000 animal taxa identified using top BLAST hits demonstrated a similar outcome^44^, highlighting the broad utility of distance-based methods for assigning unknown BINs to a higher taxonomic category. While their analysis revealed high accuracy, this result can be misleading when the ratio of positive and negative outcomes is unequal, since this can lead to a high rate of false positive errors^45^. Because our analysis generated far more correct than incorrect identifications, a precision-based metric may be preferable over ROC analysis^46^. However, thresholds estimated from the ROC-based Youden’s J statistic generated few errors (< 1%) in ordinal identification, and less than 5% at the family level. In fact, the least restrictive identification thresholds to order and family were generally estimated at P_95_ (= 5% error), an oft accepted tolerance for error in the selection of identification thresholds^17,47^. Accuracy was also highest at these thresholds but decreased as more sequences were discarded from the positive predicted class when more restrictive thresholds (i.e. P_99_, P_100_) were adopted. Thresholds with lower precision maximize the proportion of query sequences gaining an identification while limiting error rates, but those with higher precision should be adopted when misidentifications are detrimental.

Misidentifications arise when the query family is absent from the reference library or when the closest match belongs to another family, reflecting either the saturation of substitutions or incorrectly identified specimens in the reference database (but see Pentinsaari et al.^48^). In the present study, six errors of the latter type were detected and likely reflect cases where a sequence assigned to an insect order actually derived from a mite phoretic on it. However, most BINs misassigned to an order revealed no evidence of contamination. BINs assigned to an incorrect family also appeared to reflect legitimate misassignments since the reference database was strongly validated. Although the morphological identity of reference sequences derived from GenBank and those lacking vouchered specimens could not be verified, their placement within the validation tree was not suspicious. These findings demonstrate that incorrect assignments can be generated with full length sequences even at high similarity values (Order ≈ 87%, Family ≈ 91%) and that the use of short sequences increases this risk (Order ≈ 91%, Family ≈ 91%). Although some sequences from monotypic families were also incorrectly assigned with high sequence similarity (e.g., > 88%), most were discarded by the P_99_ thresholds. In fact, the highest ranked misidentifications generally corresponded with the smallest divergences observed between orders (18% p-distance, or 82% similarity) and families (10% p-distance, or 90% similarity), and were eliminated by the most restrictive thresholds.

Results generated with the most restrictive threshold (P_100_) were not affected by library completeness. However, the relationship between the maximum p-distance observed for a family and its number of component BINs suggests that low BIN coverage (< 12) often leads to underestimation of family divergence increasing the chance of misidentification. This result was supported by lower success rates, accuracy, precision, and AUC for family-level assignments in datasets with low coverage. Although thresholds estimated by Youden’s J statistic became less strict in datasets with reduced sequence coverage, precision-based thresholds became stricter and consequently less accurate. Similar declines in accuracy were reported for assignments in sphingid moths to a genus as parameterization of reference libraries was reduced^16^. Reduced library coverage was also linked to more restrictive thresholds for species-level assignments in Diptera, Hymenoptera, and Lepidoptera^47^. Collectively, these results indicate that library completeness is important for accurate barcode-based identifications^49^ even at higher ranks. Although strict thresholds can eliminate identification errors when coverage is limited, their implementation reduces the proportion of sequences identified, particularly when taxon coverage is low. Since BOLD has good coverage for most mite families common in soil and leaf litter habitats in North America, less restrictive thresholds (e.g., P_95_) could be used for the identification of mites from these settings. However, higher thresholds should be adopted for mites from other regions or from habitats (e.g., vertebrate hosts) with low coverage.

Identification success and thresholds also varied among the four orders, affirming the need for lineage-specific thresholds for higher taxon assignments. For example, 78.6% of the BINs were correctly assigned to an order using the strict similarity threshold (P_100_). However, BINs with at least 86.4% similarity to Ixodida, 83.6% to Mesostigmata, or 80.7% to Sarcoptiformes were correctly assigned to their proper order. Adopting these thresholds increased the overall proportion of BINs assigned to an order (89.2%), and a similar trend was seen at the family level. Divergent thresholds among orders likely reflect a lack of equivalency among higher ranks^50^, variation in molecular evolutionary rates^51,52^, and divergence times among lineages^53,54^. For example, the P_100_ family identification thresholds for the older yet rate accelerated acariform orders (Sarcoptiformes and Trombidiformes) were higher than for the younger parasitiform orders with more conservative rates of molecular evolution (Mesostigmata and Ixodida). In fact, all family-level identification thresholds for the Ixodida (ticks) sequences were low and reflect the minimum similarity output for this taxon since every sequence was correctly identified. Such high precision could be due the fact that just one family (Ixodidae) was evaluated, but more likely demonstrates highly successful tick identification in general since all three of the known families were present in BOLD’s library. Much higher similarity thresholds were required to generate correct family-level identifications for spiders (Arachnida: Araneae; 91%) by BLAST^17^ than for any of the mite orders in this study (75–84.6%) with the same rate of error (5%). Differences between BLAST and BOLD ID may contribute to this disparity but the combination of rank inequivalence, molecular rate variation, and divergence times are more likely culprits.

Coupling distance-based methods of higher-taxon assignment with sequence acquisition from large numbers of specimens can greatly advance understanding of mite diversity and ecology^55,56^ in a cost effective way^57,58^. Our analyses suggest that truncated sequences (e.g., 463 bp amplicon) can identify mites using BOLD ID with similar accuracy and precision as full-length DNA barcodes when thresholds were applied. Although, P_100_ thresholds did not differ significantly with sequence length, more restrictive P_99_, P_95_, and Youden’s-based thresholds were typically needed for the truncated sequences than for their full-length counterparts. A similar decline in order and family-level identification success using a naive Bayesian classifier was reported for DNA barcode sequences reduced to 400 bp^44^. However, much sharper declines in identification success were observed in sequences < 200 bp^44^ mirroring the asymptotic relationship observed between sequence length and taxonomic resolution at lower ranks^59^. Consequently, other amplicons derived from the same region as the 463 bp fragment simulated in this study should provide similar rates of success as reported here, but shorter sequences will require stricter discrimination thresholds.

## Conclusion

This study demonstrates that both full-length and truncated DNA barcodes often allow the accurate assignment of newly encountered BINs of Canadian mites to a family and order using BOLD’s identification engine (BOLD ID). However, it also highlights the need for lineage-specific thresholds to ensure the success of taxonomic assignments and reveals the sensitivity of these thresholds to both completeness of the reference library and to the length of sequences being compared. Identification accuracy and precision will certainly improve as reference libraries expand; taxa with low or no coverage should be prioritized in expanding the reference library.

## Supplementary Information

Supplementary Information.

Supplementary File S1.

Supplementary File S2.

Supplementary File S3.

Supplementary File S4.

Supplementary File S5.

Supplementary File S6.

Supplementary Tables.

## Data Availability

The aligned sequences for each of the six datasets are provided as Supplemental Files (S1–S6) while the original specimen and sequence data are available in three BOLD datasets: (1) BIN sequences: DS-BINFL; 10.5883/DS-BINFL. (2) DIV5 sequences: DS-5FLR; 10.5883/DS-5FLR. (3) DIV10 sequences: DS-10FLR; 10.5883/DS-10FLR. Current and historical reference databases used by the BOLD ID identification tool are available at https://www.boldsystems.org/index.php/IDS_OpenIdEngine.
