# Supplementary material for: DNA barcodes enable higher taxonomic assignments in the Acari
Source: Sci Rep. 2021 Aug 5;11:15922. doi: 10.1038/s41598-021-95147-8 (PMC8342613; doi:10.1038/s41598-021-95147-8)
Supplement: Supplementary file 1 — Supplementary Information. [file 41598_2021_95147_MOESM1_ESM.docx]

# Supplementary Information

**Supplementary Table S1:** Breakdown of the number of families and sequences included in datasets with three levels of sequence coverage: Barcode Index Numbers (BIN), 5% sequence clusters (DIV5), and 10% sequence clusters (DIV10). Data is summarised for all taxa combined and for each order separately.

**Supplementary Table S2:** Linear, logarithmic, and asymptotic models of the relationship between maximum intrafamilial DNA barcode divergences and the number of full-length (x̅ = 647 bp) and trimmed (x̅ = 453 bp) sequences sampled from representatives of 7,021 BINs and 162 families of mites. Estimates of model significance (p-values) and fit, including residual standard error (RSE) and Akaike information criterion (AIC), are provided.

**Supplementary Table S3:** Evaluation of the differences in the proportion of full-length (x̅ = 647 bp) and trimmed (x̅ = 453 bp) DNA barcode sequences correctly identified by BOLD ID for datasets with three levels of sequence coverage: Barcode Index Numbers (BIN), 5% sequence clusters (DIV5), and 10% sequence clusters (DIV10). Comparisons were made between sequence lengths, levels of sequence coverage, and the four orders using Pearson’s Chi-square tests. The comparison for each test is indicated by italic font with the variables ordered respectively to the counts shown for correct and incorrect identifications. Significant p-values (<0.05) are indicated with bold font.

**Supplementary Table S4:** Evaluation of the differences in the area under the ROC curves for the ordinal and family-level identifications of full-length (x̅ = 647 bp) and trimmed (x̅ = 453 bp) DNA barcodes by BOLD ID for datasets with three levels of sequence coverage: Barcode Index Numbers (BIN), 5% sequence clusters (DIV5), and 10% sequence clusters (DIV10). Comparisons of sequence lengths, levels of sequence coverage, and the four orders were generated using the method (DeLong et al. 1998) implemented by the ‘pROC’ package in R. The comparison for each test is indicated by italic font with the variables ordered respectively to the reported AUC values. Significant p-values (<0.05) are indicated with bold font.

**Supplementary Table S5:** Confusion matrix data for the order- and family-level identifications based on full-length (x̅ = 647 bp) and trimmed (x̅ = 453 bp) DNA barcodes by BOLD ID. Thresholds were estimated using Youden’s J statistic and three precision-based criteria allowing 0% (P_100_), 1% (P_99_) and 5% (P_95_) error in accepted identifications. Data is summarised for all taxa combined and for each order separately for datasets with three levels of sequence coverage: Barcode Index Numbers (BIN), 5% sequence clusters (DIV5), and 10% sequence clusters (DIV10).

**Supplementary File S1:** Alignment of 7,021 (x̅ = 647 bp) DNA barcode sequences from 189 families and four orders of mites included in the full-length BIN dataset.

**Supplementary File S2:** Alignment of 5,182 (x̅ = 647 bp) DNA barcode sequences from 189 families and four orders of mites included in the full length 5% cluster dataset (DIV5).

**Supplementary File S3:** Alignment of 3,948 (x̅ = 647 bp) DNA barcode sequences from 189 families and four orders of mites included in the full length 10% cluster dataset (DIV10).

**Supplementary File S4:** Alignment of 7,021 (x̅ = 453 bp) DNA barcode sequences from 189 families and four orders of mites included in the trimmed BIN dataset (tBIN).

**Supplementary File S5:** Alignment of 5,182 (x̅ = 453 bp) DNA barcode sequences from 189 families and four orders of mites included in the trimmed 5% cluster dataset (tDIV5).

**Supplementary File S6:** Alignment of 3,948 (x̅ = 453 bp) DNA barcode sequences from 189 families and four orders of mites included in the trimmed 10% cluster dataset (tDIV10).
